# Predictors and their prognostic value for no ROSC and mortality after a non-cardiac surgery intraoperative cardiac arrest: a retrospective cohort study

**DOI:** 10.1038/s41598-019-51557-3

**Published:** 2019-10-18

**Authors:** Matheus F. Vane, Maria J. C. Carmona, Sergio M. Pereira, Karl B. Kern, Sérgio Timerman, Guilherme Perez, Luiz Antonio Vane, Denise Aya Otsuki, José O. C. Auler Jr

**Affiliations:** 1 Discipline of Anesthesiology (LIM-08), Hospital das Clinicas HCFMUSP, Faculdade de Medicina, Universidade de São Paulo, Sao Paulo, Brazil; 2 Saver Heart Center, University of Arizona, Tucson, Arizona USA; 3 Heart Institute, Hospital das Clinicas HCFMUSP, Faculdade de Medicina, Universidade de São Paulo, Sao Paulo, Brazil; 4 Faculty of Medical Sciences of Sao Jose dos Campos – HUMANITAS, São José dos Campos, Brazil; 5 Divisao de Pneumologia, Instituto do Coracao, Hospital das Clinicas HCFMUSP, Faculdade de Medicina, Universidade de São Paulo, São Paulo, Brazil

**Keywords:** Prognostic markers, Risk factors

## Abstract

Data on predictors of intraoperative cardiac arrest (ICA) outcomes are scarce in the literature. This study analysed predictors of poor outcome and their prognostic value after an ICA. Clinical and laboratory data before and 24 hours (h) after ICA were analysed as predictors for no return of spontaneous circulation (ROSC) and 24 h and 1-year mortality. Receiver operating characteristic curves for each predictor and sensitivity, specificity, positive and negative likelihood ratios, and post-test probability were calculated. A total of 167,574 anaesthetic procedures were performed, including 158 cases of ICAs. Based on the predictors for no ROSC, a threshold of 13 minutes of ICA yielded the highest area under curve (AUC) (0.867[0.80–0.93]), with a sensitivity and specificity of 78.4% [69.6–86.3%] and 89.3% [80.4–96.4%], respectively. For the 1-year mortality, the GCS without the verbal component 24 h after an ICA had the highest AUC (0.616 [0.792–0.956]), with a sensitivity of 79.3% [65.5–93.1%] and specificity of 86.1 [74.4–95.4]. ICA duration and GCS 24 h after the event had the best prognostic value for no ROSC and 1-year mortality. For 24 h mortality, no predictors had prognostic value.

## Introduction

Intraoperative cardiac arrest (ICA) is one of the most devastating events that can occur during anaesthesia. ICA has a wide range of incidence (1–44 cases/10,000 anaesthesia) and a mortality of over 70%^1–6^.

Due to the high mortality associated with ICA, analysing predictors of poor outcomes are of great interest. The severity of organ dysfunction, cardiac arrest (CA) initial rhythm, use of epinephrine during cardiopulmonary resuscitation (CPR), previous neurological status, patient age, ICA duration, and the location of the hospital where the CA occurred have been linked to mortality^7,8^. In addition, only one study has evaluated laboratory data as a possible predictor of mortality. This study used the Physiological and Operative Severity Score for the enUmeration of Mortality and morbidity (POSSUM) and the Portsmouth predictor equation (P-POSSUM) to predict mortality in patients who had ICA^9^. However, the POSSUM includes only limited laboratory data and the study did not provide further evaluation, especially after the first 24 hours (h)^10,11^. Finally, these studies have not evaluated how precise these factors are in predicting patient mortality.

Based on these premises, our aim was to investigate the predictors and their prognostic value for no return of spontaneous circulation (ROSC) and24 h and 1-year mortality of patients who suffered ICA.

## Methods

### Inclusion and exclusion criteria

After obtaining approval from the Ethical Committee of the Clinics Hospital, Faculty of Medicine, University of Sao Paulo, Brazil (N° 0822/06) and performing the research in accordance with the Declaration of Helsinki^12^, we retrospectively reviewed the medical records of patients who were subjected to anaesthetic procedures between 2007 and 2014. The Ethical Committee waived the need for informed written consent, since it was a retrospective study. All adult patients (>18 years old) from the Central Institute of the Clinics Hospital of the University of Sao Paulo who suffered ICA were included in the analysis. ICA was defined as the absence of a central pulse associated with chest compressions for more than 10 seconds, as documented in patient medical records^13,14^. ROSC was defined as restoration of a spontaneous perfusing rhythm or arterial waveform for more than 20 minutes^15^. The intraoperative period was defined as the time between the patient´s entrance to the operating room (OR) and the patient’s exit from the OR.

The study included all elective, urgent/emergent trauma and emergent/urgent non-trauma cases, except cardiac surgery cases. Patients excluded from the study were those who were deceased organ donors, who arrived at the OR in CA, whose medical records were unavailable, and who had had cardiac surgeries.

### Analysed variables

The data were obtained from institutional patient medical records and from the laboratory system. The acquired parameters included data regarding patient status, surgery, laboratory exams and ICA (Table 1). Based on the unpredictability of the event, the data at admission, before CA, during CA, and immediately after ROSC were obtained from routine institutional records.

**Table 1:** Acquired parameters.

	Patient and Surgery	OR admission*	Pre-CA intraoperative**	Intraoperative CA	Immediately after ROSC^&^*	24h after ROSC^&^**	
**Clinical Data**	Gender (Male/Female)	Presence of hypotension (Yes or No)	Vasoactive drug usage (Yes or No)	Cause of CA		Glasgow Coma Scale (≥ 14 or ≥ 10T or <14 or 10T)	
	Age (<50 or ≥ 50 years)	Consciousness level (Sedated or Awaked)	Presence of arrhythmias (Yes or No)	Defibrillation (Yes or No)			
	ASA-PS (I, II, III, IV, or V)		Respiratory monitoring changes (Yes or No)	Initial Rhythm (Shockable, pulseless or asystole)			
	Event Shift (Daytime or Nightime)		Cardiovascular monitoring changes (Yes or No)	CA duration (minutes)			
	Type of Surgery (Elective, Trauma or Non-Trauma)			Number of epinephrine doses			
**Laboratorial Data**		PT/INR (<1.2)	Arterial pH (7.35–7.45)		Arterial pH (7.35–7.45)	Arterial pH (7.35–7.45)	PT/INR (<1.2)
		aTTP/R (<1.2)	Arterial Bicarbonate (22–26 mEq/L)		Arterial Bicarbonate (22–26 mEq/L)	Arterial Bicarbonate (22–26 mEq/L)	aTTP/R (<1.2)
		Platelet count (140–450,000 platelets/mm³)	Arterial Base Excess (−3 - +3)		Arterial Base Excess (−3 - +3)	Arterial Base Excess (−3 - +3)	Platelet count (140–450,000 platelets/mm³)
		Serum creatinine (<1.2 mg/dL)	Sodium levels (135–145 mEq/L)		Sodium levels (135–145 mEq/L)	Sodium levels (135–145 mEq/L)	Serum creatinine (<1.2)
		Serum Urea (10–50 mg/dL))	Potassium levels (3.5–5 mEq/L)		Potassium levels (3.5–5 mEq/L)	Potassium levels (3.5–5 mEq/L)	Serum Urea (10–50)
			Chloride levels (98–107 mEq/L)		Chloride levels (98–107 mEq/L)	Chloride levels (98–107 mEq/L)	
			Ionic calcium levels (4.4–5.4 mEq/L)		Ionic calcium levels (4.4–5.4 mEq/L)	Ionic calcium levels (4.4–5.4 mEq/L)	
			Arterial Lactate (<14.4 mg/dL)		Arterial Lactate (<14.4 mg/dL)	Arterial Lactate (<14.4 mg/dL)	
			Glucose levels (70–100 mg/dL)		Glucose levels (70–100 mg/dL)	Glucose levels Glucose levels (70–100 mg/dL)	
			Hemoglobin (12–16 g/dL)		Hemoglobin (12–16 g/dL)	Hemoglobin (12–16 g/dL)	
			Hematocrit (35–47%)		Hematocrit (35–47%)	Hematocrit (35–47%)	

Variable categorization in parenthesis. aTTP/R: normalized ratio of activated partial thromboplastin time; ASA-PS: American Society of Anesthesiologists Physical Statuts Classification; PT/INR: international normalized ratio of prothrombin time; ROSC: Return of spontaneous circulation.

*Tolerance: 24 h for elective cases and 6 h for urgent/emergent cases; **Tolerance: Clinical data: 60 min before CA; Laboratorial data: 120 minutes before CA; & *Tolerance: 60 min & **Tolerance: 6 h.

Hypotension: Systolic Arterial Pressure <90 mmHg or mean arterial pressure <50 mmHg; Arrhythmias: Any new ECG findings or rhythm changes; Respiratory Monitoring Changes: new findings on oximetry, capnography, airway pressure, or tidal volume; Cardiovascular Monitoring Changes: new findings on heart rate or blood pressure.

### Outcomes (no ROSC, 24 h mortality and 1-year mortality)

The patients were evaluated for no ROSC and 24 h and 1-year mortality after the event. We contacted the patients after discharge by phone calls, emails, and text messages to account for mortality.

### 2.5 Statistical analysis

Data were collected and managed using REDCap electronic data capture software hosted at the institution^16^. Data were analysed by using the statistical software R 3.5.1 (R Foundation for Statistical Computing, Vienna, Austria) and RStudio (RStudio Team, Boston, MA). The library “pROC”^17^ was used for the ROC analysis in R. STATA (StataCorp LP, College Station, Texas, USA) software was also used to obtain the graphics for this paper.

Considering the high prevalence found in this study for each outcome, the authors estimated the prevalence ratio (PR) and its respective 95% confidence interval (95% CI) from bivariate analysis based on the outcome of each variable. To perform the binomial analysis, continuous and discrete variables were categorized as presented in Table 1. From the binomial analysis, variables that had p values lower than 0.20 were selected for multivariate analysis using Poisson regression with robust variance. The variable with the lowest p value from the bivariate analysis was first selected, and then, other variables with higher p values were added to the analysis of the data. The authors retained the variables with p < 0.05 for the final model. Finally, the PR for the final model was estimated for each variable with its respective 95% CI.

After analysing the final model for each outcome, receiver operating characteristic curves (ROC) were generated for the predictors. The ROC curves were created using the bootstrap methodology (2000 samples). The areas under the ROC curves (AUCs) and confidence intervals calculated and were compared using the Delong and Clarke-Pearson method^18^. In addition, sensitivity, specificity and positive and negative likelihood ratios (LHR), along with their 95%CI were calculated. The cut-off values were chosen according to the highest Youden index, calculated as (sensitivity + specificity −1). The AUC was considered non-discriminant if the 95%CI included 0.5. For the ROC analysis of the GCS, the vocal response category was ignored to include patients who were awake and could follow commands but who were intubated or tracheostomized (i.e., a GCS of 10 T). The curve with the highest AUC was selected and compared with the other ROC curves using DeLong’s test for two correlated ROC curves. The two-step Fagan nomogram was used for post-test probability calculation^19^. Continuous variables were categorized according to the Youden index. The pre-test probability was estimated using the sample data. The significance level adopted by this study for all analyses was p < 0.05.

The survival curves for the 1-year mortality of patients who had ROSC were obtained by the Kaplan-Meier product-limit estimator.

## Results

### Sample characterization

**Figure 1:**
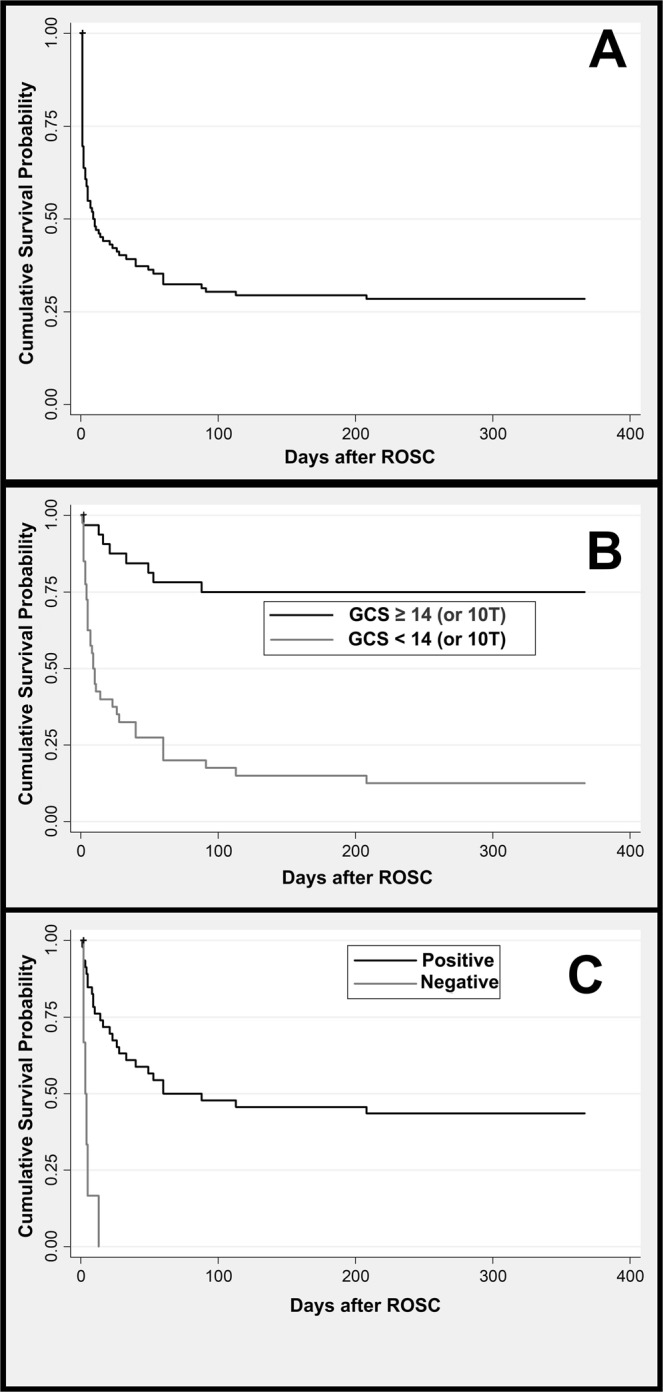
Study flowgram diagram.

There were 167,574 anaesthetic procedures (138,896 elective procedures and 28,678 urgent/emergent procedures), with 160 ICAs. Two patients (1.2%) were transferred to another institution before 24 h after ROSC and were excluded from the analyses (Fig. 1).

**Figure 2:**
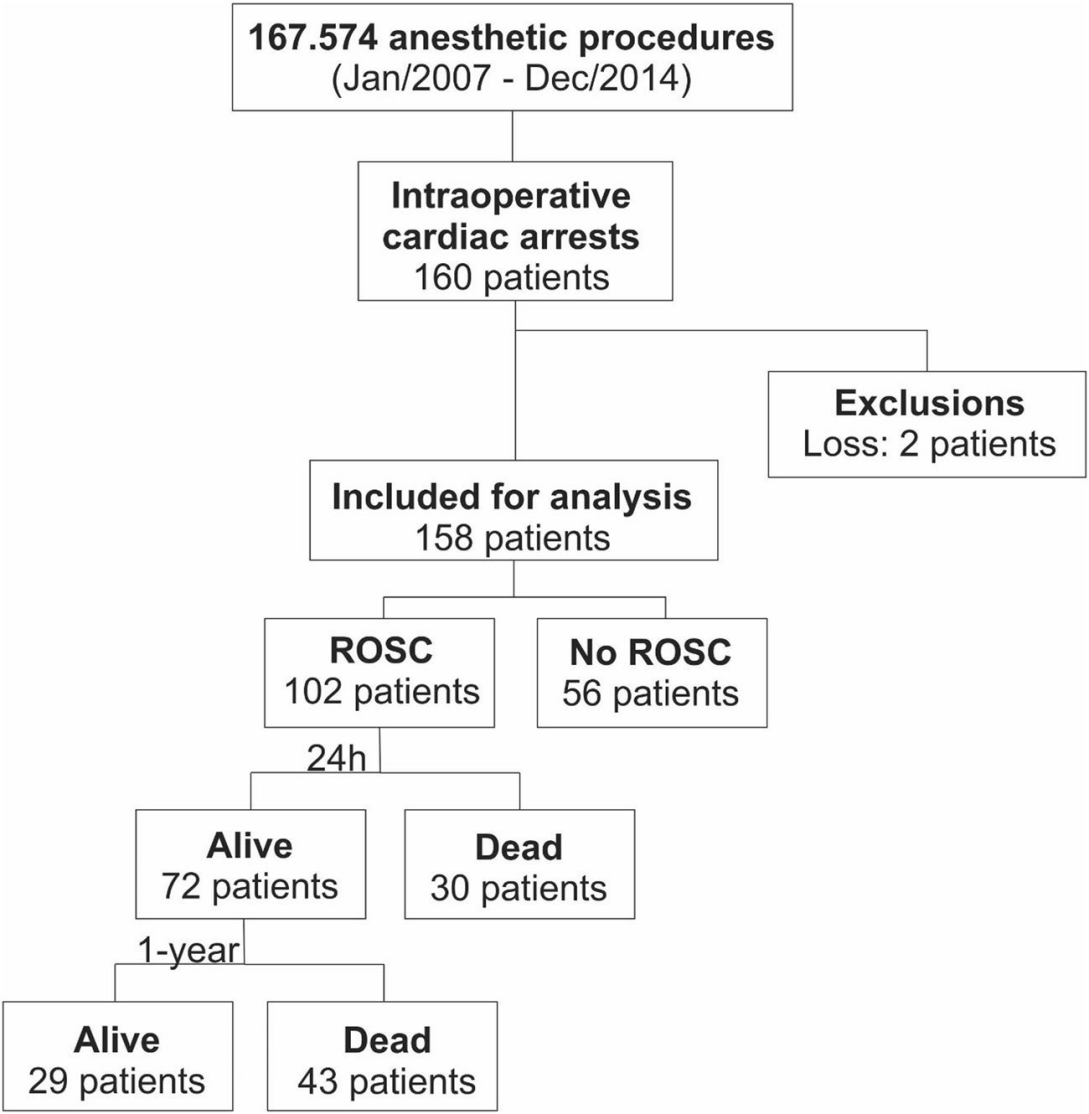
Plots of Kaplan-Meier product limit estimates of survival after ICA. (**A**): for patients with intraoperative cardiac arrest; (**B**): According to the INR/PT variation; (**C**): according to GCS 24 h after the event. +: Censoring of two cases who were lost to follow-up.

The overall ICA prevalence was 9.54 cases/10,000 anaesthesia procedures, with 41.5 cases/10.000 anaesthesia procedures for urgent/emergent cases and 2.74/10.000 anaesthesia procedures for elective surgeries. Fifty-six patients did not achieve ROSC (case-fatality rate: 35.4% [CI 28.0–43.4]). For those who achieved ROSC, 30 patients died within 24 h (case-fatality rate: 29.4% [CI 20.8–39.3]), and 73 patients died within one year (case-fatality rate: 71.6% [CI 61.8–80.1]) (Fig. 2). There were 38, 47, and 75 ICAs in elective, trauma and non-trauma patients, respectively.

The full data set is available in online supplement 1. Most of the patients included in the study were men (57.6%), with a median age of 53 [33–69.5] years and ASA-PS III (31.9%) or I (26.9%). Most of the ICA occurred during non-trauma emergency surgeries (46.1%). The median length of ICA was 11 [4.0–22.3] minutes. All cases, except for one, occurred during general anaesthesia.

For elective surgeries, the most common cause of ICA was acute myocardial infarction (26.3%). For trauma and non-trauma surgeries, the most common cause of ICA was hypovolemia (trauma: 80.9%; non-trauma: 24%).

All patients who survived for more than one year were neurologically intact with a GCS of 15, except for one patient who continued to be tracheostomized and had a GCS of 10 T.

### Predictors for no ROSC, 24 h mortality and 1-year mortality

The predictors for the analysed endpoints are shown in Table 2. The predictors for no ROSC were hypovolemia as the cause of ICA (adjusted PR 2.42 [CI 1.52–3.86], p < 0.001), hypotension at OR admission (adjusted PR 1.60 [CI 1.07–2.40], p = 0.02) and ICA duration (adjusted PR 1.03 [CI 1.02–1.04], p < 0.001).

**Table 2:** Predictors for mortality.

Predictors	PR_w_	PR_adj_	CI 95% (PR_adj_)	*p*
**No ROSC**				
*Hypovolemia as the Cause of CA*	3.36	2.42	1.52–3.86	**<0.001**
*Hypotension at OR admission*	1.85	1.60	1.07–2.40	**0.022**
*CA duration (per minute)*	1.03	1.03	1.02–1.04	**<0.001**
**24 h Mortality**				
*Hypovolemia as the Cause of CA*	3.01	4.15	1.98–8.68	**<0.001**
*Na* < *135 or* > *145 mEq/L before CA*	2.16	2.37	1.23–4.58	**0.010**
*PT/INR* > *1.2 at admission*	2.71	2.43	1.24–4.75	**0.010**
*CA duration (per minute)*	1.03	1.04	1.03–1.06	**<0.001**
**1-year Mortality**				
*Glasgow Coma Scale 24 after ROSC* < *14 ou* < 10 *T*	3.50	2.70	1.52–4.79	**0.001**
*Negative PT/INR variation between admission and the first 24* *h after ROSC*	1.29	1.66	1.11–2.47	**0.013**
*CA duration (per minute)*	1.01	1.01	1.01–1.04	**0.048**

CA: Cardiac arrest; 95% CI: 95% Confidence interval; PT/INR: international normalized ratio of prothrombin time; PR_aj_: Adjusted prevalence ratio; PR_r_: Raw prevalence ratio.

Regarding 24 h mortality, hypovolemia as the cause of ICA (adjusted PR 4.15 [95% CI 1.98–8.68], p < 0.001), sodium greater than 145 mEq/L or less than 135 mEq/L at admission (adjusted PR 2.37 [CI 1.23–4.58], p = 0.010), PT/INR greater than 1.2 at admission (adjusted PR 2.43 [CI 1.24–4.75], p = 0.010), and duration of ICA (per minute) (adjusted PR 1.04 [CI 1.03–1.06], p < 0.001) were the predictors.

The multivariate model analysis showed that the 1-year mortality was independently associated with GCS < 14 (or 10 T for intubated patients) 24 h after ROSC (adjusted PR 2.70 [95% CI 1.52–4.79], p = 0.001), a decrease in the PT/INR during the first 24 h (adjusted PR 1.66 [95% CI 1.11–2.47], p = 0.013), and the ICA duration (adjusted PR 1.01 [95% CI 1.01–1.04], p = 0.048) (Fig. 2).

### ROC analysis of the predictors

The specificity, sensitivity, area under curve (AUC) and thresholds for each predictor of mortality are shown in Table 3. The LHR and post-test probabilities are shown in Table 4. Based on the independent risk factors for no ROSC, a threshold of 13 minutes of ICA yielded a significantly higher AUC than the other independent risk factors (0.867 vs 0.737 vs 0.610, p = 0.04, p < 0.001), with a sensitivity and specificity of 78.4% [69.6–86.3%] and 89.3 [80.4–93.3%], respectively.

**Table 3:** Sensitivity, specificity and area under curve (AUC) for the predictors.

	Sensitivity		Specificity		AUC		Threshold	p-AUC
	%	95% *CI*	%	95% *CI*		95% *CI*		
No-ROSC								
*CA duration (minutes)*	78.43	69.61–86.27	89.29	80.36–96.43	0.8665	0.8004–0.9328	13.5	—
*Cause of CA*	77.50	68.63–85.29	67.90	55.36–80.36	0.7265	0.6526–0.8005	Hypovolemia	**0.004**
*Hypotension at OR admission*	77.50	68.63–85.29	44.60	32.14–57.14	0.6104	0.5332–0.6878	Presence	**<0.001**
**24 h Mortality**								
*CA duration (minutes)*	61.64	50.68–72.60	56.67	40.00–73.30	0.6159	0.4997–0.7323	10	—
*Cause of CA*	73.97	64.38–83.56	46.67	30.00–63.33	0.6032	0.4993–0.7072	Hypovolemia	0.882
*Sodium levels before CA (mEq/L)*	68.25	57.14–79.37	43.48	26.09–65.22	0.5310	0.3824–0.6797	136	0.374
*PT/INR at admission*	58.18	45.45–70.91	28.57	9.52–47.62	0.5662	0.3149–0.5526	>1.2	0.617
**1-year Mortality**								
*Glasgow Coma Scale 24 after ROSC*	79.31	65.52–93.10	86.05	74.42–95.35	0.8737	0.7918–0.9556	9*	—
*CA duration (minutes)*	64.38	53.42–75.34	79.31	65.52–93.10	0.7503	0.6482–0.8525	5.5	0.066
*PT/INR variation between admission and the first 24 h after ROSC*	18.75	6.25–34.38	1	1	0.5937	0.5251–0.6624	Negative	**<0.001**

*Glasgow Coma Scale with supression of best vocal response. Legend: CA: Cardiac arrest; 95% CI: 95% Confidence interval; OR: operating room; PT/INR: international normalized ratio of prothrombin time; ROSC: return of spontaneous circulation; p-AUC: vs the highest ROC AUC.

**Table 4:** Positive and Negative Likelihood ratios (+LHR, -LHR), post-tests probability for the analyzed thresholds.

	LHR+	95%CI	Post-test Probability	95% CI	LHR−	95% CI	Post-test Probability	95% CI
**No-ROSC**								
*CA duration (minutes)*	7.32	3.41–16	93%	86–97%	0.24	0.17–0.35	30%	24–39%
*Cause of CA*	2.41	1.62–3.58	81%	75–87%	0.33	0.22–0.50	38%	29–48%
*Hypotension at OR admission*	1.40	1.08–1.81	72%	66–77%	0.51	0.32–0.80	48%	37–59%
**24** **h Mortality**								
*CA duration (minutes)*	1.42	0.91–2.23	78%	69–84%	0.68	0.44–1.04	62%	52–72%
*Cause of CA*	1.39	0.97–1.99	77%	70–83%	0.56	0.32–0.96	58%	44–70%
*Sodium levels before CA (mEq/L)*	1.18	0.79–1.76	76%	68–83%	0.77	0.43–1.37	68%	54–79%
*PT/INR at admission*	0.81	0.57–1.16	68%	60–75%	1.46	0.7–3.08	79%	65–89%
**1-Year Mortality**								
Glasgow Coma Scale 24 after ROSC*	6.82	2.93–16	82%	66–92%	0.23	0.11–0.48	13%	7–24%
*CA duration (minutes)*	3.11	1.5–6.48	89%	79–94%	0.45	0.31–0.64	53%	44–62%
*PT/INR variation between admission and the first 24 h after ROSC*	Inf	0.49–139	100%	44–100%	0.81	0.69–0.99	56%	52–61%

*Glasgow Coma Scale with supression of best vocal response. Legend: CA: Cardiac arrest; 95% CI: 95% Confidence interval; OR: operating room; PT/INR: international normalized ratio of prothrombin time; ROSC: return of spontaneous circulation.

For the 24 h mortality, no predictors were associated with prognosis.

GCS without a vocal response 24 h after ROSC, CA duration and PT/INR variation between admission and the first 24 h after ROSC were associated with 1-year mortality. GCS without a vocal response had the highest AUC, with a sensitivity of 79.3% [65.5–93.1%] and specificity of 86.1 [74.4–95.4]. GCS without a vocal response 24 h after ROSC had a significantly higher AUC than PT/INR variation (0.874 vs 0.594, respectively, p = < 0.001).

## Discussion

The main findings of this study were that based on the predictors for no ROSC, a threshold of 13 minutes of ICA yielded a significantly higher AUC than the other predictors. For the 24 h mortality, no predictors had prognostic value. For the 1-year mortality, the GCS 24 h after ICA had the highest AUC.

The ICA duration was independently associated with no ROSC and 1-year mortality and had lower cut-offs for longer survival. Ray *et al*. proposed that good diagnostic value of a biomarker would have an accuracy of 0.75–0.90, a +LHR between 5 and 10, a -LHR between 0.1 and 0.2 and an AUC between 0.75 and 0.90^20^. An ICA duration of greater than 13 minutes predicted no ROSC. This means that if an ICA lasts for more than 13 minutes, there is a 93% probability that this patient will not achieve ROSC. Of note, the ICA duration had a direct association with the degree of hypoxia, which is predictor of poor outcome following out-of-hospital CA^21,22^. In animal studies, the average time of anoxia associated with irreversible neurologic damage is between 4 and 10 minutes^23,24^. However, in humans, when effective CPR is present, the CA duration can be more than 17 minutes, while still having a good neurological outcome^21^. One out-of-hospital CA study considered only a CA duration of greater than 25 minutes as a risk of death^22^. In contrast to previous findings, in the 1-year mortality analysis, an ICA duration of 5.5 minutes had the greatest AUC for sensitivity and specificity and wasassociated with a higher probability of death and an even lower ICA duration than previous studies^21,22^. This finding was supported by the fact that in patients who achieved ROSC, there was a 1% increase in the patient´s likelihood of death for each minute of CA according to the 1-year mortality.

Several studies linked the cause of ICA to a prognostic factor^25–28^. In our study, only hypovolemia as a cause of ICA was independently associated only with no ROSC. This is in accordance with previous studies on the association of the number of transfused units, the amount of intraoperative bleeding and hypovolemia with in-hospital mortality^26–28^. In addition, these studies showed that the cause of ICA was no longer associated with 30-day mortality, which is in accordance with our findings. One may assume that after the first 24 h, hypovolemia is most likely resolved, and organ damage (ischaemia-reperfusion injury) is mainly associated with the outcome. Furthermore, the fact that the ICA duration has a better prognostic value than the cause of ICA for mortality might suggest that organ damage is more time-dependent than aetiology-dependent.

The use of GCS after CA has been linked to neurological prognosis. The higher the GCS is 24 h after CA, the better the neurological outcome^29,30^. It is estimated that 80% of patients will be comatose after CA^31^. Within 24 h, more than 95% of patients who recover consciousness will awake^31^. Within 48 h, if targeted temperature management is present, 78% of patients who recover consciousness will awake^32^. We found that GCS without a verbal response 24 h after the event was a predictor of 1-year mortality, with a good AUC, a high + LHR and a low -LHR, yielding a positive post-test probability of 82% and a negative post-test probability of 13%. Hence, we can assume that if the value of the GCS is lower than 15 or 10 T after 24 h, there is an 82% probability of 1-year mortality for the patient. Thus, this variable can be classified as having good prognostic value for mortality^20^. Eventually, since all patients who survived for one year had a GCS of 15 or 10 T, we could also infer that these conclusions, drawn for mortality, could be extrapolated for neurological outcomes.

To the best of our knowledge, this is the first study to evaluate laboratory and clinical data at admission, immediately before ICA, immediately after ROSC, and 24 h after ROSC. This study was also the first to analyse the association of changes in laboratory data during the first 24 h with the 1-year mortality and to include a sensitivity and specificity analysis of each of these variables.

Regarding laboratory data, PT/INR at admission was independently associated with a greater risk of death within 24 h and its increase in the first 24 h was associated with 1-year mortality. In this manner, we could presume that PT/INR should be analysed as a static and dynamic variable. During acidosis, hypothermia, and/or haemodilution, which are common situations for patients experiencing or recovering from CA, the coagulation system is greatly affected, resulting in hypocoagulation^33^. Recently, hypocoagulation has been attributed to tissue hypoperfusion, with a direct association between the degree of hypoperfusion and the intensity of changes in the coagulation system^34^. In a previous study, base excess levels lower than −6 were associated with increased PT and aTTP, reinforcing the theory that hypoperfusion can generate coagulopathy^35^. In this study, most of the patients had base excess lower than −6, either before or after the event, which is a possible explanation for the increase in PT/INR. Although the PT/INR variation had a high specificity and an infinite +LHR for 1-year mortality, the sensitivity was low, resulting in a small AUC and low −LHR. Lactate, which is another marker of hypoperfusion, did not correlate with the outcomes. This might be because most of the analysed patients (95.6%) had high lactate levels after CA and no patient with normal lactate levels died.

Finally, a surprising result of our study is that, contrary to previous studies, the ASA-PS and the comorbidities were neither independently associated nor had any prognostic value with any of the analysed outcomes. The inclusion of trauma patients, who were mostly ASA-PS I and II, might explain this lack of association^2,25,36,37^. The high number of trauma patients might have biased the analysis of ASA-PS as a predictor.

### Limitations

This study had some limitations. First, we pooled and compared all surgeries — elective, urgent and emergent. This was done due to the low prevalence of ICA, especially in elective surgeries. Thus, the sample in this study consisted of more urgent and emergent surgeries than elective surgeries. This fact, however, allowed greater external validity of the study, especially for tertiary academic hospitals, where there is a mixture of elective, urgent and emergent surgeries. In addition, we could not differentiate between the total number of procedures performed for trauma and non-trauma surgeries due to the lack of an electronic medical record. Second, this was a single-centred study performed at a tertiary academic hospital where the patients are clinically more severe, which might have increased the risk of death. The advantage of using a single centred is that one can assume standardized patient care, including the management of CPR. Third, this was a retrospective study based on patients’ charts. There might be some record bias that we were not aware of. Some anaesthesiologists might have underestimated the duration of the ICA, while others might have misdiagnosed the cause or the electrical rhythm. Fourth, another important limitation is the decision to continue resuscitative efforts during cardiac arrest. A longer ICA duration (especially if longer than 20 minutes) is more prone to termination of resuscitative efforts^38^. In our retrospective study, the treating clinicians were not blinded to the ICA duration at the time of the ICA, and the predictive estimates may be artificially inflated.

Although we had some loss of follow-up, it was very low. Most of the patients either died during hospitalization or continued to visit the hospital for routine follow-up, the loss to follow-up was limited. In addition, institutional electronic system can log every time the patient comes for a consultation, allowing the authors to check for survival. Finally, we acknowledge the limitations caused by the wide confidence intervals of some of the analyses, the large number of factors analysed and thepoor calibration and discrimination of some models.

## Conclusion

ICA duration and GCS 24 h after the event had the best prognostic value for ROSC and 1-year mortality. For 24 h mortality, no predictors had prognostic value. Larger, multicentred studies should be performed to validate these findings.

## Supplementary information

Supplementary Dataset 1
