# Supplementary material for: Predictors and their prognostic value for no ROSC and mortality after a non-cardiac surgery intraoperative cardiac arrest: a retrospective cohort study
Source: Sci Rep. 2019 Oct 18;9:14975. doi: 10.1038/s41598-019-51557-3 (PMC6802384; doi:10.1038/s41598-019-51557-3)
Supplement: Supplementary file 1 — Supplementary Dataset 1 [file 41598_2019_51557_MOESM1_ESM.docx]

**Predictors and their prognostic value for no ROSC and mortality after a non-cardiac surgery intraoperative cardiac arrest: a retrospective cohort study**

Matheus F. Vane; Maria J. C. Carmona; Sergio M. Pereira; Karl B. Kern; Sérgio Timerman; Guilherme Perez; Luiz Antonio Vane; Denise Aya Otsuki; José O. C. Auler Jr.

**Suplement 1**: Full dataset including binomial analysis of the analyzed variables. ASA-PS: American Society of Anesthesiologists Physical Statuts Classification; aTTP/R: normalized ratio of activated partial thromboplastin time; CA: Cardiac arrest; CI 95%: Confidence interval 95%; OR: operating room; PT/INR: international normalized ratio of prothrombin time; RP: Prevalence ratio; ROSC: return of spontaneous circulation

| Variables | Total | No ROSC | | RR | IC 95% (RR) | *p* |  | Total | 24h Mortality | | PR | CI 95% (PR) | *p* |  | Total | 1-year mortality | | PR | CI 95% (PR) | *p* |  |
| --- | --- | --- | --- | --- | --- | --- | --- | --- | --- | --- | --- | --- | --- | --- | --- | --- | --- | --- | --- | --- | --- |
|  | **n^o^** | **n^o^** | **%** |  |  |  |  | **n^o^** | **n^o^** | **%** |  |  |  |  | **n^o^** | **n^o^** | **%** |  |  |  |  |
| Gender |  |  |  |  |  | 0.363 |  |  |  |  |  |  | 0.28 |  |  |  |  |  |  | 0.172 |  |
| Male | 91 | 35 | 38.5 | 1 |  |  |  | 56 | 19 | 33.9 | 1 |  |  |  | 56 | 37 | 66.1 | 1 |  |  |  |
| Female | 67 | 21 | 31.3 | 0.81 | 0.52 - 1.27 |  |  | 46 | 11 | 23.9 | 0.7 | 0.37 - 1.33 | |  | 46 | 36 | 78.3 | 1.18 | 0.93 - 1.51 |  |  |
| Age (years) | |  |  |  |  | 0.730 |  |  |  |  |  |  | 0.108 |  |  |  |  |  |  | 0.168 |  |
| < 50 | 73 | 25 | 34.2 | 1 |  |  |  | 48 | 10 | 20.8 | 1 |  |  |  | 48 | 31 | 64.6 | 1 |  |  |  |
| ≥ 50 | 84 | 31 | 36.9 | 1.08 | 0.70 - 1.65 |  |  | 53 | 19 | 35.8 | 1.72 | 0.89 - 3.34 | |  | 53 | 41 | 77.4 | 1.2 | 0.93 - 1.55 |  |  |
| ASA-PS* |  |  |  |  |  | 0.991^a^ |  |  |  |  |  |  | 0.662^a^ |  |  |  |  |  |  | 0.081^a^ |  |
| I | 43 | 18 | 41.9 | 1 |  |  |  | 25 | 9 | 36 | 1 |  |  |  | 25 | 18 | 72 | 1 |  |  |  |
| II | 20 | 8 | 40.0 | 0.96 | 0.50 - 1.82 |  |  | 12 | 1 | 8.3 | 0.23 | 0.33 - 1.64 | |  | 12 | 4 | 33.3 | 0.46 | 0.20 - 1.07 |  |  |
| III | 51 | 11 | 21.6 | 0.51 | 0.27 - 0.97 |  |  | 40 | 10 | 25 | 0.69 | 0.33 - 1.47 | |  | 40 | 29 | 72.5 | 1.01 | 0.74 - 1.38 |  |  |
| IV | 34 | 11 | 32.4 | 0.77 | 0.42 - 1.41 |  |  | 23 | 9 | 39.1 | 1.09 | 0.52 - 2.26 | |  | 23 | 20 | 87 | 1.21 | 0.90 - 1.62 |  |  |
| V | 9 | 7 | 77.8 | 1.86 | 1.13 - 3.06 |  |  | 2 | 1 | 50 | 1.39 | 0.31 - 6.15 | |  | 2 | 2 | 100 | 1.39 | 1.09 - 1.78 |  |  |
| Event Shift | |  |  |  |  | 0.409 |  |  |  |  |  |  | 0.292 |  |  |  |  |  |  | 0.796 |  |
| Daytime | 108 | 36 | 33.3 | 1 |  |  |  | 72 | 19 | 26.4 | 1 |  |  |  | 72 | 51 | 70.8 | 1 |  |  |  |
| Nightime | 50 | 20 | 40.0 | 1.20 | 0.78 - 1.85 |  |  | 30 | 11 | 36.7 | 1.39 | 0.75 – 2.56 | |  | 30 | 22 | 73.3 | 1.04 | 0.80 - 1.35 |  |  |
| Type of Surgery | |  |  |  |  | 0.650 |  |  |  |  |  |  | 0.362 |  |  |  |  |  |  | **0.019** |  |
| Elective | 38 | 8 | 21.1 | 1 |  |  |  | 30 | 7 | 23.3 | 1 |  |  |  | 30 | 16 | 53.3 | 1 |  |  |  |
| Non-trauma | 73 | 26 | 35.6 | 1.69 | 0.85 - 3.38 |  |  | 47 | 13 | 27.7 | 1.19 | 0.53 - 2.64 | |  | 47 | 40 | 85.1 | 1.6 | 1.12 - 2.28 |  |  |
| Trauma | 47 | 22 | 46.8 | 2.22 | 1.12 - 4.43 |  |  | 25 | 10 | 40 | 1.71 | 0.76 - 3.86 | |  | 25 | 17 | 68 | 1.28 | 0.83 - 1.96 |  |  |
| Presence of hypotension at OR admission | | | | |  | **0.003** |  |  |  |  |  |  | **0.021** |  |  |  |  |  |  | 0.38 |  |
| No | 110 | 31 | 28.2 | 1 |  |  |  | 79 | 19 | 24.1 | 1 |  |  |  | 79 | 55 | 69.6 | 1 |  |  |  |
| Yes | 48 | 25 | 52.1 | 1.85 | 1.23 - 2.77 |  |  | 23 | 11 | 47.8 | 1.99 | 1.11 - 3.56 | |  | 23 | 18 | 78.3 | 1.12 | 0.87 - 1.46 |  |  |
| Vasoactive drug usage before CA | | | | |  | **0.018** |  |  |  |  |  |  | **0.001** |  |  |  |  |  |  | **0.005** |  |
| No | 80 | 21 | 26.3 | 1 |  |  |  | 59 | 9 | 15.3 | 1 |  |  |  | 59 | 36 | 61 | 1 |  |  |  |
| Yes | 78 | 35 | 44.9 | 1.71 | 1.10 - 2.66 |  |  | 43 | 21 | 48.8 | 3.2 | 1.63 - 6.31 | |  | 43 | 37 | 86 | 1.41 | 1.11 - 1.79 |  |  |
| Consciousness level | | |  |  |  | 0.086 |  |  |  |  |  |  | 0.088 |  |  |  |  |  |  | 0.348 |  |
| Sedated | 62 | 27 | 43.5 | 1 |  |  |  | 35 | 14 | 40 | 1 |  |  |  | 35 | 27 | 77.1 | 1 |  |  |  |
| Awaked | 96 | 29 | 30.2 | 0.69 | 0.46 - 1.05 |  |  | 67 | 16 | 23.9 | 0.6 | 0.33 - 1.08 | |  | 67 | 46 | 68.7 | 0.89 | 0.70 - 1.14 |  |  |
| Arrhythmia | |  |  |  |  | 0.545 |  |  |  |  |  |  | 0.545 |  |  |  |  |  |  | 0.857 |  |
| No | 69 | 19 | 27.5 | 1 |  |  |  | 69 | 19 | 27.5 | 1 |  |  |  | 69 | 49 | 71 | 1 |  |  |  |
| Yes | 33 | 11 | 33.3 | 1.21 | 0.65 - 2.25 |  |  | 33 | 11 | 33.3 | 1.21 | 0.65 - 2.25 | |  | 33 | 24 | 72.7 | 1.02 | 0.79 - 1.33 |  |  |
| Respiratory monitoring changes | | | | | | |  |  |  |  |  |  | 0.791 |  |  |  |  |  |  | 0.914 |  |
| No | 94 | 28 | 29.8 | 1 |  |  |  | 66 | 20 | 30.3 | 1 |  |  |  | 66 | 47 | 71.2 | 1 |  |  |  |
| Yes | 64 | 28 | 43.8 | 1.47 | 0.97 - 2.23 |  |  | 36 | 10 | 27.8 | 0.92 | 0.48 - 1.75 | |  | 36 | 26 | 72.2 | 1.01 | 0.79 - 1.31 |  |  |
| Cardiovascular monitoring changes | | | | | | |  |  |  |  |  |  | 0.818 |  |  |  |  |  |  | 0.357 |  |
| No | 64 | 18 | 28.1 | 1 |  |  |  | 46 | 13 | 28.3 | 1 |  |  |  | 46 | 35 | 76.1 | 1 |  |  |  |
| Yes | 94 | 38 | 40.4 | 1.53 | 0.90 - 2.29 |  |  | 56 | 17 | 30.4 | 1.07 | 0.58 - 1.98 | |  | 56 | 38 | 67.9 | 0.89 | 0.70 - 1.14 |  |  |
| pH before CA | | | |  |  | 0.162 |  |  |  |  |  |  | 0.128 |  |  |  |  |  |  | 0.498 |  |
| 7.35 – 7.45 | 28 | 7 | 25.0 | 1 |  |  |  | 21 | 4 | 19 | 1 |  |  |  | 21 | 15 | 71.4 | 1 |  |  |  |
| <7.35 / >7.45 | 98 | 40 | 40.8 | 1.63 | 0.82 - 3.25 |  |  | 58 | 23 | 39.7 | 2.08 | 0.81 - 5.35 | |  | 58 | 46 | 79.3 | 1.11 | 0.82 - 1.50 |  |  |
| Bicarbonate before CA (mEq/L) | | | | | | 0.696 |  |  |  |  |  |  | 0.22 |  |  |  |  |  |  | 0.385 |  |
| 22 - 26 | 18 | 6 | 33.3 | 1 |  |  |  | 12 | 2 | 16.7 | 1 |  |  |  | 12 | 8 | 66.7 | 1 |  |  |  |
| <22 / >26 | 107 | 41 | 38.3 | 1.15 | 0.57 - 2.31 |  |  | 66 | 25 | 37.9 | 2.27 | 0.61 - 8.43 | |  | 66 | 53 | 80.3 | 1.2 | 0.79 - 1.83 |  |  |
| Base Excess before CA | | | | |  | 0.304 |  |  |  |  |  |  | 0.138 |  |  |  |  |  |  | 0.777 |  |
| -3 - +3 | 21 | 10 | 47.6 | 1 |  |  |  | 11 | 1 | 9.1 | 1 |  |  |  | 11 | 8 | 72.7 | 1 |  |  |  |
| <-3 / > +3 | 102 | 37 | 36.3 | 0.76 | 0.45 - 1.28 |  |  | 65 | 25 | 38.5 | 4.23 | 0.63 - 28.47 | |  | 65 | 50 | 76.9 | 1.06 | 0.72 - 1.56 |  |  |
| Sodium levels before CA (mEql/L) | | | |  |  | 0.865 |  |  |  |  |  |  | **0.037** |  |  |  |  |  |  | 0.756 |  |
| 135 – 145 | 81 | 31 | 38.3 | 1 |  |  |  | 50 | 9 | 18 | 1 |  |  |  | 50 | 36 | 72 | 1 |  |  |  |
| <135 / >145 | 57 | 21 | 36.8 | 0.96 | 0.62 - 1.49 |  |  | 36 | 14 | 38.9 | 2.16 | 1.05 - 4.46 | |  | 36 | 27 | 75 | 1.04 | 0.81 - 1.35 |  |  |
| Potassium levels before CA (mEq/L) | | | |  |  | 0.713 |  |  |  |  |  |  | 0.163 |  |  |  |  |  |  | 0.125 |  |
| 3.5 – 5 | 84 | 33 | 39.3 | 1 |  |  |  | 51 | 11 | 21.6 | 1 |  |  |  | 51 | 34 | 66.7 | 1 |  |  |  |
| < 3.5 / >5 | 58 | 21 | 36.2 | 0.92 | 0.60 - 1.42 |  |  | 37 | 13 | 35.1 | 1.63 | 0.82 - 3.24 | |  | 37 | 30 | 81.1 | 1.22 | 0.95 - 1.56 |  |  |
| Chloride levels before CA (mEq/L) | | | |  |  | 0.213 |  |  |  |  |  |  | 0.986 |  |  |  |  |  |  | 0.494 |  |
| 98 - 107 | 39 | 13 | 33.3 | 1 |  |  |  | 26 | 8 | 30.8 | 1 |  |  |  | 26 | 22 | 84.6 | 1 |  |  |  |
| <98 / >107 | 67 | 31 | 46.3 | 1.39 | 0.83 - 2.33 |  |  | 36 | 11 | 30.6 | 0.99 | 0.46 - 2.13 | |  | 36 | 28 | 77.8 | 0.92 | 0.72 - 1.17 |  |  |
| Ionic calcium levels (mg/dL) | | | |  |  | **0.046** |  |  |  |  |  |  | 0.151 |  |  |  |  |  |  | 0.939 |  |
| 4.4 – 5.4 | 36 | 9 | 25.0 | 1 |  |  |  | 27 | 5 | 18.5 | 1 |  |  |  | 27 | 21 | 77.8 | 1 |  |  |  |
| <4.4 / > 5.4 | 79 | 37 | 46.8 | 1.87 | 1.01 - 3.47 |  |  | 42 | 15 | 35.7 | 1.93 | 0.79 - 4.72 | |  | 42 | 33 | 78.6 | 1.01 | 0.78 - 1.31 |  |  |
| Lactate before CA (mg/dL) | | | |  |  | 0.157 |  |  |  |  |  |  | 0.261 |  |  |  |  |  |  | 0.515 |  |
| ≤ 14.4 | 17 | 4 | 23.5 | 1 |  |  |  | 13 | 2 | 15.4 | 1 |  |  |  | 13 | 11 | 84.6 | 1 |  |  |  |
| > 14.4 | 87 | 39 | 44.8 | 1.91 | 0.78 - 4.65 |  |  | 48 | 16 | 33.3 | 2.17 | 0.56 - 8.33 | |  | 48 | 37 | 77.1 | 0.91 | 0.69 - 1.21 |  |  |
| Glucose levels before CA (mg/dL) | | | |  |  | 0.712 |  |  |  |  |  |  | 0.148 |  |  |  |  |  |  | 0.091 |  |
| 70 – 100 | 19 | 7 | 36.8 | 1 |  |  |  | 12 | 1 | 8.3 | 1 |  |  |  | 12 | 6 | 50 | 1 |  |  |  |
| < 70 / > 70 | 89 | 37 | 41.6 | 1.13 | 0.59 - 2.14 |  |  | 52 | 18 | 34.6 | 4.15 | 0.60 - 28.57 | |  | 52 | 43 | 82.7 | 1.65 | 0.92 - 2.97 |  |  |
| Hemoglobin before CA (mg/dL) | | | |  |  | 0.171 |  |  |  |  |  |  | 0.764 |  |  |  |  |  |  | 0.407 |  |
| 12 – 16 | 31 | 8 | 25.8 | 1 |  |  |  | 23 | 6 | 26.1 | 1 |  |  |  | 23 | 15 | 65.2 | 1 |  |  |  |
| <12 / >16 | 114 | 46 | 40.4 | 1.56 | 0.82 - 2.96 |  |  | 68 | 20 | 29.4 | 1.13 | 0.51 - 2.47 | |  | 68 | 51 | 75 | 1.15 | 0.83 - 1.60 |  |  |
| Hematocrit before CA (%) | | | |  |  | 0.256 |  |  |  |  |  |  | 0.562 |  |  |  |  |  |  | 0.582 |  |
| 35 – 47 | 40 | 12 | 30.0 | 1 |  |  |  | 28 | 9 | 32.1 | 1 |  |  |  | 28 | 19 | 67.9 | 1 |  |  |  |
| < 35 / > 47 | 103 | 42 | 40.8 | 1.36 | 0.80 - 2.31 |  |  | 61 | 16 | 26.2 | 0.82 | 0.41 - 1.62 | |  | 61 | 45 | 73.8 | 1.09 | 0.81 - 1.46 |  |  |
| PT/INR at admission | | |  |  |  | 0.148 |  |  |  |  |  |  | **0.02** |  |  |  |  |  |  | **0.005** |  |
| <1.2 | 54 | 15 | 27.8 | 1 |  |  |  | 39 | 6 | 15.4 | 1 |  |  |  | 39 | 23 | 59 | 1 |  |  |  |
| >1.2 | 61 | 25 | 41.0 | 1.48 | 0.87 - 2.50 |  |  | 36 | 15 | 41.7 | 2.71 | 1.17 - 6.25 | |  | 36 | 32 | 88.9 | 1.51 | 1.13 - 2.01 |  |  |
| aTTP/R at admission | | |  |  |  | 0.198 |  |  |  |  |  |  | 0.205 |  |  |  |  |  |  | 0.143 |  |
| <1.2 | 74 | 23 | 31.1 | 1 |  |  |  | 51 | 12 | 23.5 | 1 |  |  |  | 51 | 35 | 68.6 | 1 |  |  |  |
| >1.2 | 42 | 18 | 42.9 | 1.38 | 0.85 - 2.25 |  |  | 24 | 9 | 37.5 | 1.59 | 0.78 - 3.27 | |  | 24 | 10 | 41.7 | 1.21 | 0.94 - 1.57 |  |  |
| Platelet count (platelets/mm³) | | | | |  | 0.188 |  |  |  |  |  |  | 0.059 |  |  |  |  |  |  | 0.087 |  |
| 140.000 – 450.000 | 91 | 28 | 30.8 | 1 |  |  |  | 63 | 15 | 23.8 | 1 |  |  |  | 63 | 43 | 68.3 | 1 |  |  |  |
| <140.000 / >450.000 | 35 | 15 | 42.9 | 1.39 | 0.85 - 2.28 |  |  | 20 | 9 | 45 | 1.89 | 0.98 - 3.66 | |  | 20 | 17 | 85 | 1.25 | 0.97 - 1.60 |  |  |
| Creatinine at admission (mg/dL) | | | |  |  | 0.459 |  |  |  |  |  |  | 0.397 |  |  |  |  |  |  | 0.392 |  |
| <1.2 | 62 | 19 | 30.6 | 1 |  |  |  | 43 | 11 | 25.6 | 1 |  |  |  | 43 | 30 | 69.8 | 1 |  |  |  |
| >1.2 | 65 | 24 | 36.9 | 1.2 | 0.74 - 1.97 |  |  | 41 | 14 | 34.1 | 1.33 | 0.68 - 2.60 | |  | 41 | 32 | 78 | 1.12 | 0.87 - 1.45 |  |  |
| Urea at admission (mg/dL) | | | |  |  | 0.142 |  |  |  |  |  |  | 0.671 |  |  |  |  |  |  | 0.379 |  |
| 10 - 50 | 61 | 17 | 27.9 | 1 |  |  |  | 44 | 12 | 27.3 | 1 |  |  |  | 44 | 31 | 70.5 | 1 |  |  |  |
| <10 / >50 | 64 | 26 | 40.6 | 1.46 | 0.88 - 2.41 |  |  | 38 | 12 | 31.6 | 1.16 | 0.59 - 2.28 | |  | 38 | 30 | 78.9 | 1.12 | 0.87 - 1.44 |  |  |
| Cause of CA | | | |  |  | **<0.001** |  |  |  |  |  |  | **<0.001** |  |  |  |  |  |  | 0.124 |  |
| Others | 97 | 18 | 18.6 | 1 |  |  |  | 79 | 16 | 20.3 | 1 |  |  |  | 79 | 54 | 68.4 | 1 |  |  |  |
| Hypovolemia | 61 | 38 | 62.3 | 3.36 | 2.12 - 5.33 |  |  | 23 | 14 | 60.9 | 3.01 | 1.74 - 5.21 | |  | 23 | 19 | 82.6 | 1.21 | 0.95 - 1.54 |  |  |
| Defibrilation during CA | | | |  |  | 0.205 |  |  |  |  |  |  | 0.194 |  |  |  |  |  |  | 0.279 |  |
| No | 129 | 48 | 37.2 | 1 |  |  |  | 81 | 21 | 25.9 | 1 |  |  |  | 81 | 56 | 69.1 | 1 |  |  |  |
| Yes | 26 | 6 | 23.1 | 0.62 | 0.30 - 1.30 |  |  | 20 | 8 | 40 | 1.54 | 0.80 - 2.97 | |  | 20 | 16 | 80 | 1.16 | 0.89 - 1.51 |  |  |
| Rhythm of CA | |  |  |  |  | 0.739 |  |  |  |  |  |  | 0.945 |  |  |  |  |  |  | 0.687 |  |
| Shockable | 25 | 7 | 28.0 | 1 |  |  |  | 18 | 5 | 27.8 | 1 |  |  |  | 18 | 13 | 72.2 | 1 |  |  |  |
| Pulseless cardiac arrest | 40 | 15 | 37.5 | 1.34 | 0.63 - 2.83 |  |  | 25 | 7 | 28 | 1.01 | 0.38 - 2.68 | |  | 25 | 16 | 64 | 0.89 | 0.59 - 1.34 |  |  |
| Asystole | 90 | 32 | 35.6 | 1.27 | 0.64 - 2.53 |  |  | 58 | 18 | 31 | 1.12 | 0.48 - 2.59 | |  | 58 | 43 | 74.1 | 1.03 | 0.74 - 1.42 |  |  |
| pH immediately after ROSC | | | | |  |  |  |  |  |  |  |  | 0.315 |  |  |  |  |  |  | 0.191 |  |
| 7.35 – 7.45 | |  |  |  |  |  |  | 9 | 1 | 11.1 | 1 |  |  |  | 9 | 4 | 44.4 | 1 |  |  |  |
| <7.35 / >7.45 | |  |  |  |  |  |  | 89 | 26 | 29.2 | 2.63 | 0.40 - 17.33 | |  | 89 | 65 | 73 | 1.64 | 0.78 - 3.46 |  |  |
| pCO_2_ immediately after ROSC (mmHg) | | | |  |  |  |  |  |  |  |  |  | 0.604 |  |  |  |  |  |  | 0.161 |  |
| 35 – 45 |  |  |  |  |  |  |  | 29 | 7 | 24.1 | 1 |  |  |  | 29 | 23 | 79.3 | 1 |  |  |  |
| <35 / >45 |  |  |  |  |  |  |  | 68 | 20 | 29.4 | 1.22 | 0.58 - 2.57 | |  | 68 | 45 | 66.2 | 0.83 | 0.65 - 1.07 |  |  |
| Bicarbonate immediately after ROSC (mEq/L) | | | | |  |  |  |  |  |  |  |  | 0.129 |  |  |  |  |  |  | 0.958 |  |
| 22 - 26 |  |  |  |  |  |  |  | 14 | 1 | 7.1 | 1 |  |  |  | 14 | 10 | 71.4 | 1 |  |  |  |
| <22 / >26 |  |  |  |  |  |  |  | 82 | 26 | 31.7 | 4.44 | 0.65 - 30.44 | |  | 82 | 58 | 70.7 | 0.99 | 0.69 - 1.42 |  |  |
| Base excess immediately after ROSC | | | |  |  |  |  |  |  |  |  |  | 0.264 |  |  |  |  |  |  | 0.883 |  |
| -3 - +3 |  |  |  |  |  |  |  | 14 | 2 | 14.3 | 1 |  |  |  | 14 | 10 | 71.4 | 1 |  |  |  |
| <-3 / >+3 |  |  |  |  |  |  |  | 82 | 25 | 30.5 | 2.13 | 0.56 - 8.08 | |  | 82 | 57 | 69.5 | 0.97 | 0.68 - 1.40 |  |  |
| Sodium levels immediately after ROSC (mEq/L) | | | | |  |  |  |  |  |  |  |  | **0.019** |  |  |  |  |  |  | 0.104 |  |
| 135 – 145 |  |  |  |  |  |  |  | 60 | 12 | 20 | 1 |  |  |  | 60 | 39 | 65 | 1 |  |  |  |
| <135 / >145 | |  |  |  |  |  |  | 35 | 15 | 42.9 | 2.14 | 1.13 - 4.05 | |  | 35 | 28 | 80 | 1.23 | 0.96 - 1.58 |  |  |
| Potassium levels immediately after ROSC (mEq/L) | | | | |  |  |  |  |  |  |  |  | 0.932 |  |  |  |  |  |  | 0.34 |  |
| 3.5 – 5 |  |  |  |  |  |  |  | 51 | 14 | 27.5 | 1 |  |  |  | 51 | 34 | 66.7 | 1 |  |  |  |
| < 3.5 / >5 |  |  |  |  |  |  |  | 45 | 12 | 26.7 | 0.97 | 0.50 - 1.88 | |  | 45 | 34 | 75.6 | 1.13 | 0.88 - 1.47 |  |  |
| Chloride levels immediately after ROSC (mEq/L) | | | | |  |  |  |  |  |  |  |  | 0.295 |  |  |  |  |  |  | 0.314 |  |
| 98 – 107 |  |  |  |  |  |  |  | 37 | 13 | 35.1 | 1 |  |  |  | 37 | 25 | 67.6 | 1 |  |  |  |
| <98 / >107 | |  |  |  |  |  |  | 45 | 11 | 24.4 | 0.7 | 0.35 - 1.37 | |  | 45 | 35 | 77.8 | 1.15 | 0.88 - 1.51 |  |  |
| Ionic calcium levels immediately after ROSC (mg/dL) | | | | | |  |  |  |  |  |  |  | **0.026** |  |  |  |  |  |  | 0.079 |  |
| 4.4 – 5.4 |  |  |  |  |  |  |  | 37 | 5 | 13.5 | 1 |  |  |  | 37 | 23 | 62.2 | 1 |  |  |  |
| <4.4 / > 5.4 | |  |  |  |  |  |  | 51 | 19 | 37.3 | 2.76 | 1.13 - 6.74 | |  | 51 | 41 | 80.4 | 1.29 | 0.97 - 1.72 |  |  |
| Lactate immediately after ROSC (mg/dL) | | | | |  |  |  |  |  |  |  |  | NP |  |  |  |  |  |  | 0.87 |  |
| ≤ 14.4 |  |  |  |  |  |  |  | 4 | 0 | 0 |  |  |  |  | 4 | 3 | 75 | 1 |  |  |  |
| > 14.4 |  |  |  |  |  |  |  | 84 | 23 | 27.4 |  |  |  |  | 84 | 60 | 71.4 | 0.95 | 0.53 - 1.71 |  |  |
| Hemoglobin immediately after ROSC (mg/dL) | | | | |  |  |  |  |  |  |  |  | 0.169 |  |  |  |  |  |  | 0.258 |  |
| 12 – 16 |  |  |  |  |  |  |  | 13 | 1 | 7.7 | 1 |  |  |  | 13 | 7 | 53.8 | 1 |  |  |  |
| <12 / >16 |  |  |  |  |  |  |  | 81 | 24 | 29.6 | 3.85 | 0.56 - 26.35 | |  | 81 | 59 | 72.8 | 1.35 | 0.80 - 2.28 |  |  |
| Hematocrit immediately after ROSC (%) | | | |  |  |  |  |  |  |  |  |  | 0.387 |  |  |  |  |  |  | 0.611 |  |
| 35 – 47 |  |  |  |  |  |  |  | 17 | 3 | 17.6 | 1 |  |  |  | 17 | 11 | 64.7 | 1 |  |  |  |
| < 35 / > 47 | |  |  |  |  |  |  | 77 | 22 | 28.6 | 1.62 | 0.54 - 4.82 | |  | 77 | 55 | 71.4 | 1.1 | 0.75 - 1.61 |  |  |
| pH variation before CA and after ROSC | | | |  |  |  |  |  |  |  |  |  | 0.254 |  |  |  |  |  |  | 0.9 |  |
| Positive |  |  |  |  |  |  |  | 22 | 9 | 40.9 | 1 |  |  |  | 22 | 17 | 77.3 | 1 |  |  |  |
| Negative |  |  |  |  |  |  |  | 54 | 15 | 27.8 | 0.68 | 0.35 - 1.32 | |  | 54 | 41 | 75.9 | 0.98 | 0.75 - 1.29 |  |  |
| Bicarbonate variation before CA and after ROSC | | | | |  |  |  |  |  |  |  |  | NP |  |  |  |  |  |  | NP |  |
| Negative |  |  |  |  |  |  |  | 50 |  |  |  |  |  |  | 50 | 33 | 66 |  |  |  |  |
| Positive |  |  |  |  |  |  |  | 0 |  |  |  |  |  |  | 0 |  |  |  |  |  |  |
| Lactate variation before CA and after ROSC | | | | |  |  |  |  |  |  |  |  | 0.975 |  |  |  |  |  |  | 0.825 |  |
| Positive |  |  |  |  |  |  |  | 47 | 12 | 25.5 | 1 |  |  |  | 47 | 37 | 78.7 | 1 |  |  |  |
| Negative |  |  |  |  |  |  |  | 8 | 2 | 25 | 0.98 | 0.26 - 3.62 | |  | 8 | 6 | 75 | 0.95 | 0.62 - 1.47 |  |  |
| Potassium variation before CA and after ROSC | | | | |  |  |  |  |  |  |  |  | 0.831 |  |  |  |  |  |  | 0.309 |  |
| Positive |  |  |  |  |  |  |  | 48 | 13 | 27.1 | 1 |  |  |  | 48 | 37 | 77.1 | 1 |  |  |  |
| Negative |  |  |  |  |  |  |  | 36 | 9 | 25 | 0.92 | 0.44 - 1.93 | |  | 36 | 24 | 66.7 | 0.86 | 0.65 - 1.14 |  |  |
| Chloride variation before CA and after ROSC | | | | |  |  |  |  |  |  |  |  | NP |  |  |  |  |  |  | 0.135 |  |
| Positive |  |  |  |  |  |  |  | 24 | 0 |  |  |  |  |  | 24 | 20 | 83.3 | 1 |  |  |  |
| Negative |  |  |  |  |  |  |  | 14 | 1 |  |  |  |  |  | 14 | 8 | 57.1 | 0.69 | 0.42 - 1.12 |  |  |
| Ionic calcium variation before CA and after ROSC | | | | |  |  |  |  |  |  |  |  | NP |  |  |  |  |  |  | 0.741 |  |
| Positive |  |  |  |  |  |  |  | 31 | 1 |  |  |  |  |  | 31 | 21 | 67.7 | 1 |  |  |  |
| Negative |  |  |  |  |  |  |  | 18 | 0 |  |  |  |  |  | 18 | 13 | 72.2 | 1.07 | 0.73 - 1.56 |  |  |
| CA duration (per minute) | | |  | 1.03 | 1.02 - 1.04 | **<0.001** |  |  |  |  | 1.03 | 1.02 - 1.04 | **<0.001** |  |  |  |  | 1.01 | 1.00 - 1.02 | **<0.001** |  |
| Number of epinephrine doses (per dose) | | | | 1.18 | 1.02 - 1.07 | **<0.001** |  |  |  |  | 1.18 | 1.09 - 1.28 | **<0.001** |  |  |  |  | 1.07 | 1.03 – 1.11 | **0.001** |  |
| Glasgow Coma Scale 24h after ROSC | | | |  |  |  |  |  |  |  |  |  |  |  |  |  |  |  |  | **<0.001** |  |
| ≥ 14 or ≥ 10T | |  |  |  |  |  |  |  |  |  |  |  |  |  | 32 | 8 | 25.0 | 1 |  |  |  |
| < 14 or < 10T | |  |  |  |  |  |  |  |  |  |  |  |  |  | 40 | 35 | 87.5 | 3.50 | 1.89 - 6.48 |  |  |
| pH 24h after ROSC | |  |  |  |  |  |  |  |  |  |  |  |  |  |  |  |  |  |  | 0.306 |  |
| 7.35 – 7.45 | |  |  |  |  |  |  |  |  |  |  |  |  |  | 30 | 16 | 53.3 | 1 |  |  |  |
| <7.35 / >7.45 | |  |  |  |  |  |  |  |  |  |  |  |  |  | 41 | 27 | 65.9 | 1.23 | 0.82 - 1.85 |  |  |
| pCO_2_ 24h after ROSC (mmHg) | | |  |  |  |  |  |  |  |  |  |  |  |  |  |  |  |  |  | 0.455 |  |
| 35 – 45 |  |  |  |  |  |  |  |  |  |  |  |  |  |  | 29 | 16 | 55.2 | 1 |  |  |  |
| <35 / >45 |  |  |  |  |  |  |  |  |  |  |  |  |  |  | 42 | 27 | 64.3 | 1.17 | 0.78 - 1.74 |  |  |
| Bicarbonate levels 24h after ROSC (mEq/L) | | | | |  |  |  |  |  |  |  |  |  |  |  |  |  |  |  | 0.623 |  |
| 22 – 26 |  |  |  |  |  |  |  |  |  |  |  |  |  |  | 20 | 13 | 65.0 | 1 |  |  |  |
| <22 / >26 |  |  |  |  |  |  |  |  |  |  |  |  |  |  | 51 | 30 | 58.8 | 0.90 | 0.61 - 1.35 |  |  |
| Sodium levels 24h after ROSC (mEq/L) | | | |  |  |  |  |  |  |  |  |  |  |  |  |  |  |  |  | 0.271 |  |
| 135 – 145 |  |  |  |  |  |  |  |  |  |  |  |  |  |  | 39 | 21 | 53.8 | 1 |  |  |  |
| <135 / >145 | |  |  |  |  |  |  |  |  |  |  |  |  |  | 33 | 22 | 66.7 | 1.24 | 0.85 - 1.81 |  |  |
| Potassium levels 24h after ROSC | | | |  |  |  |  |  |  |  |  |  |  |  |  |  |  |  |  | 0.536 |  |
| 3.5 – 5 |  |  |  |  |  |  |  |  |  |  |  |  |  |  | 34 | 19 | 55.9 | 1 |  |  |  |
| < 3.5 / >5 |  |  |  |  |  |  |  |  |  |  |  |  |  |  | 38 | 24 | 63.2 | 1.13 | 0.77 - 1.67 |  |  |
| Chloride levels 24h after ROSC (mEq/L) | | | |  |  |  |  |  |  |  |  |  |  |  |  |  |  |  |  | 0.311 |  |
| 98 - 107 |  |  |  |  |  |  |  |  |  |  |  |  |  |  | 19 | 10 | 52.6 | 1 |  |  |  |
| <98 / >107 | |  |  |  |  |  |  |  |  |  |  |  |  |  | 40 | 27 | 67.5 | 1.28 | 0.79- 2.08 |  |  |
| Ionic calcium 24h after ROSC (mg/dL) | | | |  |  |  |  |  |  |  |  |  |  |  |  |  |  |  |  | 0.253 |  |
| 4.4 – 5.4 |  |  |  |  |  |  |  |  |  |  |  |  |  |  | 30 | 16 | 53.3 | 1 |  |  |  |
| <4.4 / > 5.4 | |  |  |  |  |  |  |  |  |  |  |  |  |  | 37 | 25 | 67.6 | 1.27 | 0.84 - 1.90 |  |  |
| Lactate 24h after ROSC (mg/dL) | | | |  |  |  |  |  |  |  |  |  |  |  |  |  |  |  |  | 0.419 |  |
| ≤ 14.4 |  |  |  |  |  |  |  |  |  |  |  |  |  |  | 11 | 5 | 45.5 | 1 |  |  |  |
| > 14.4 |  |  |  |  |  |  |  |  |  |  |  |  |  |  | 53 | 32 | 60.4 | 1.33 | 0.67 - 2.64 |  |  |
| Hemoglobin 24h after ROSC (mg/dL) | | | |  |  |  |  |  |  |  |  |  |  |  |  |  |  |  |  | 0.140 |  |
| 12 – 16 |  |  |  |  |  |  |  |  |  |  |  |  |  |  | 15 | 6 | 40.0 | 1 |  |  |  |
| <12 / >16 |  |  |  |  |  |  |  |  |  |  |  |  |  |  | 55 | 36 | 65.5 | 1.64 | 0.85 - 3.15 |  |  |
| Hematocrit 24h after ROSC (%) | | | |  |  |  |  |  |  |  |  |  |  |  |  |  |  |  |  | 0.267 |  |
| 35 – 47 |  |  |  |  |  |  |  |  |  |  |  |  |  |  | 17 | 8 | 47.1 | 1 |  |  |  |
| < 35 / > 47 | |  |  |  |  |  |  |  |  |  |  |  |  |  | 53 | 34 | 64.2 | 1.36 | 0.79 - 2.36 |  |  |
| PT/INR 24h after ROSC | | |  |  |  |  |  |  |  |  |  |  |  |  |  |  |  |  |  | 0.380 |  |
| ≤ 1.2 |  |  |  |  |  |  |  |  |  |  |  |  |  |  | 23 | 12 | 52.2 | 1 |  |  |  |
| > 1.2 |  |  |  |  |  |  |  |  |  |  |  |  |  |  | 47 | 30 | 63.8 | 1.22 | 0.78 - 1.92 |  |  |
| aTTP/R 24h after ROSC | | |  |  |  |  |  |  |  |  |  |  |  |  |  |  |  |  |  | 0.500 |  |
| ≤ 1.2 |  |  |  |  |  |  |  |  |  |  |  |  |  |  | 34 | 19 | 55.9 | 1 |  |  |  |
| > 1.2 |  |  |  |  |  |  |  |  |  |  |  |  |  |  | 36 | 23 | 63.9 | 1.14 | 0.77 - 1.69 |  |  |
| Platelet count 24h after ROSC (plaquetas/mm³) | | | | |  |  |  |  |  |  |  |  |  |  |  |  |  |  |  | 0.174 |  |
| 140.000 – 450.000 | |  |  |  |  |  |  |  |  |  |  |  |  |  | 32 | 16 | 50.0 | 1 |  |  |  |
| <140.000 / >450.000 | |  |  |  |  |  |  |  |  |  |  |  |  |  | 39 | 26 | 66.7 | 1.33 | 0.88 - 2.02 |  |  |
| Creatinine 24h after ROSC (mg/dL) | | | |  |  |  |  |  |  |  |  |  |  |  |  |  |  |  |  | 0.464 |  |
| ≤ 1.2 |  |  |  |  |  |  |  |  |  |  |  |  |  |  | 26 | 14 | 53.8 | 1 |  |  |  |
| > 1.2 |  |  |  |  |  |  |  |  |  |  |  |  |  |  | 46 | 29 | 63.0 | 1.17 | 0.77 - 1.79 |  |  |
| Urea 24h after ROSC (mg/dL) | | |  |  |  |  |  |  |  |  |  |  |  |  |  |  |  |  |  | 0.670 |  |
| 10 – 50 |  |  |  |  |  |  |  |  |  |  |  |  |  |  | 37 | 21 | 56.8 | 1 |  |  |  |
| <10 / >50 |  |  |  |  |  |  |  |  |  |  |  |  |  |  | 34 | 21 | 61.8 | 1.09 | 0.74 - 1.61 |  |  |
| PT/INR variation between admission and the first 24h after ROSC | | | | | | |  |  |  |  |  |  |  |  |  |  |  |  |  | **<0.001** |  |
| Positive | |  |  |  |  |  |  |  |  |  |  |  |  |  | 46 | 26 | 56.5 | 1 |  |  |  |
| Negative | |  |  |  |  |  |  |  |  |  |  |  |  |  | 6 | 6 | 100 | 1.77 | 1.37 - 2.29 |  |  |
| aTTP/R variation between admission and the first 24h after ROSC | | | | | | |  |  |  |  |  |  |  |  |  |  |  |  |  | 0.229 |  |
| Positive | |  |  |  |  |  |  |  |  |  |  |  |  |  | 34 | 19 | 55.9 | 1 |  |  |  |
| Negative | |  |  |  |  |  |  |  |  |  |  |  |  |  | 18 | 13 | 72.2 | 1.29 | 0.85 - 1.96 |  |  |
| Platelet count variation between admission and the first 24h after ROSC | | | | | | |  |  |  |  |  |  |  |  |  |  |  |  |  | 0.920 |  |
| Positive | |  |  |  |  |  |  |  |  |  |  |  |  |  | 13 | 8 | 61.5 | 1 |  |  |  |
| Negative | |  |  |  |  |  |  |  |  |  |  |  |  |  | 45 | 27 | 60.0 | 0.98 | 0.595 - 1.60 |  |  |
| Creatinine variation between admission and the first 24h after ROSC | | | | | | |  |  |  |  |  |  |  |  |  |  |  |  |  | 0.910 |  |
| Positive | |  |  |  |  |  |  |  |  |  |  |  |  |  | 37 | 23 | 62.2 | 1 |  |  |  |
| Negative | |  |  |  |  |  |  |  |  |  |  |  |  |  | 22 | 14 | 63.6 | 1.02 | 0.68 - 1.54 |  |  |
| Urea variation between admission and the first 24h after ROSC | | | | | | |  |  |  |  |  |  |  |  |  |  |  |  |  | 0.771 |  |
| Positive | |  |  |  |  |  |  |  |  |  |  |  |  |  | 12 | 8 | 66.7 | 1 |  |  |  |
| Negative | |  |  |  |  |  |  |  |  |  |  |  |  |  | 45 | 28 | 62.2 | 0.93 | 0.59 - 1.48 |  |  |
